# Ectopic thyroid in the hepatoduodenal ligament: a case report and literature review

**DOI:** 10.3389/fonc.2024.1378885

**Published:** 2024-04-22

**Authors:** Lei Zhang, Xijun Cui, Baolei Wang, Xiulan Du, Guoqi Hou, Xiaoqian Yu

**Affiliations:** ^1^ Department of Hepatobiliary Surgery, Qingdao University Affiliated Weihai Central Hospital, Weihai, Shandong, China; ^2^ Department of General Surgery, Weihai Hospital of Traditional Chinese Medicine, Weihai, Shandong, China; ^3^ Department of General Surgery, People’s Hospital of LongKou City, Yantai, Shandong, China; ^4^ Meical Section, Qingdao University Affiliated Weihai Central Hospital, Weihai, Shandong, China; ^5^ Department of Pathology, Qingdao University Affiliated Weihai Central Hospital, Weihai, Shandong, China; ^6^ Department of Obstetrics, Qingdao University Affiliated Weihai Central Hospital, Weihai, Shandong, China

**Keywords:** ectopic thyroid, hepatoduodenal ligament, extra thyroid gland, case report, vagal thyroid

## Abstract

Ectopic thyroid arises from abnormal development of thyroid primordial tissues as it migrates to the lower interstitium during the embryonic period, which can occur at various locations during the descent process. However, ectopic thyroid in the subdiaphragmatic area is extremely rare. In this case, we report a case of ectopic thyroid located in the hepatoduodenal ligament. The 60-year-old female patient was admitted to hospital with gallbladder stones and cholecystitis. Preoperative imaging showed a mass in the hepatoduodenal ligament. As the patient declined a needle biopsy of the mass, the nature of the mass remained unclear prior to surgery. The patient subsequently underwent laparoscopic cholecystectomy and exploratory resection of the mass. The histopathology of the resected mass showed the characteristics of ectopic thyroid, and immunohistochemical staining revealed positive expression of thyroid transcription factor-1 and thyroglobulin. The diagnosis of ectopic thyroid was established. Upon confirming the diagnosis, comprehensive neck examination revealed the presence of a normally functioning thyroid gland. Throughout the four-year follow-up period, the patient’s thyroid ultrasonography and thyroid function tests indicated no abnormalities. Ectopic thyroid in the hepatoduodenal ligament and surrounding areas is an extremely rare clinical abnormality, achieving a clear diagnosis before initiating treatment offers diagnostic and treatment insights and clues for clinicians when differentiating masses within this region.

## Introduction

Ectopic thyroid is a developmental disease, which is caused by abnormal development of the thyroid primordial tissues during migration to the lower interstitium in the embryonic period. Ectopic thyroid can occur at different locations, but typically it is found along the midline of the base of the tongue and the diaphragm. Most cases are located in the base of the tongue, but they can also be found in the laryngotrachea, esophagus, lungs, mediastinum and chest cavity. Locations distant from the submandibular area, particularly within the abdominal cavity, are uncommon or rare (1, 2). Additional research indicates that intra-abdominal ectopic thyroid might also result from a shared embryological origin, where the thyroid shares foregut endoderm with the gastrointestinal tract, pancreas and liver (3, 4).

The clinical literature on abdominal thyroid ectopia is limited, which may indicate that most patients with this condition are asymptomatic and cases are discovered incidentally. Clinicians may also misdiagnose ectopic thyroid as a tumor, cyst or lipoma (5). Herein, we report a rare case of ectopic thyroid located in the hepatoduodenal ligament. The patient refused to undergo fine-needle aspiration biopsy before surgery and therefore we could not make a clear diagnosis. Given the surgical indication for removal of gallbladder stones combined with cholecystitis, the mass was excised during laparoscopic cholecystectomy. Postoperative pathology confirmed the mass to be an ectopic thyroid, and postoperative examination verified the existence of normal thyroid in the neck with normal function. Hence, it is crucial to diagnose ectopic thyroid accurately prior to initiating treatment. Through the review and synthesis of pertinent literature, this case aimed to enhance medical professionals’ understanding of the condition, provide valuable information for diagnosis and treatment, and minimize the risks of misdiagnosis and inappropriate treatment.

## Case presentation

**Figure 1:**
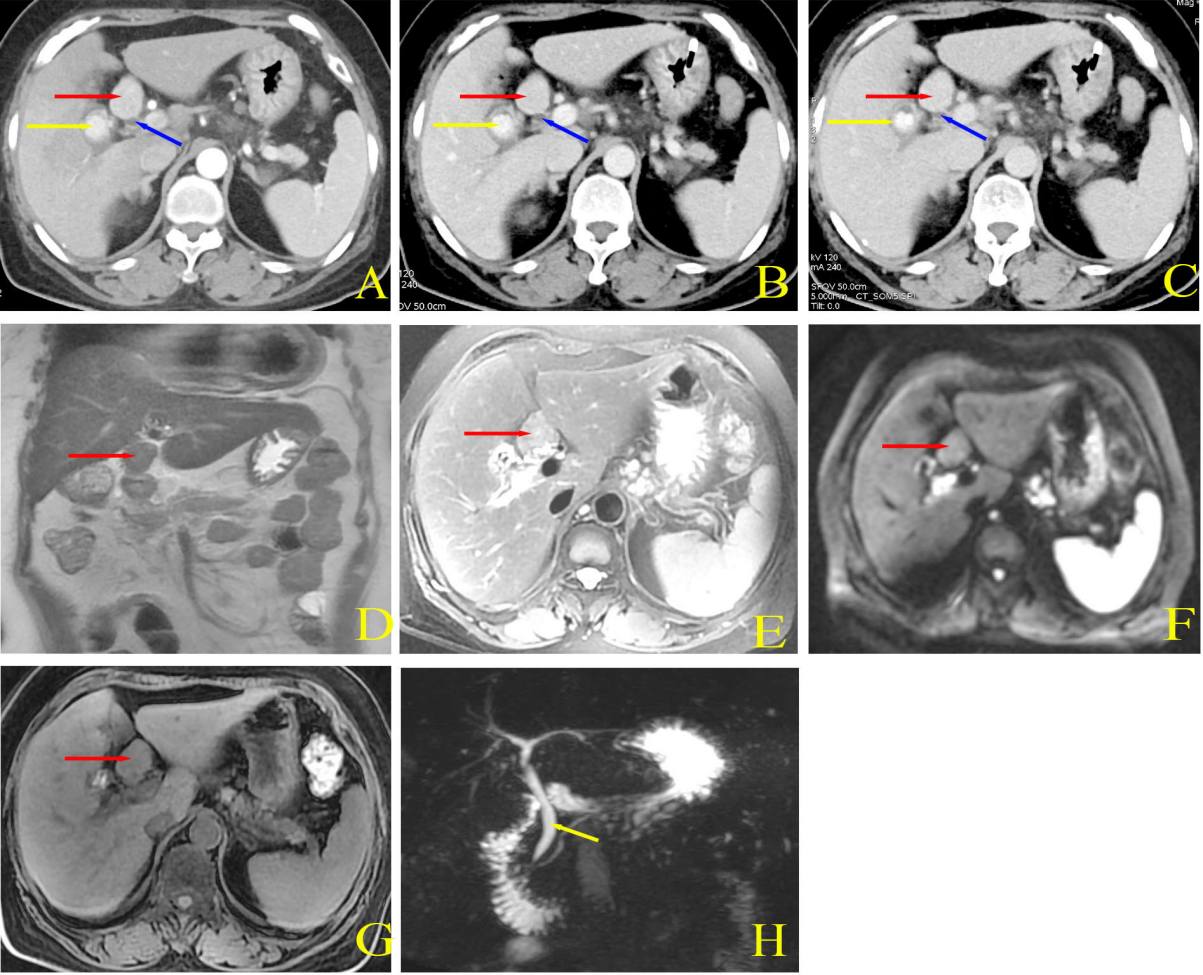
Preoperative abdominal CT imaging. **(A)** Arterial phase demonstrated ovoid mass with clear borders and markedly homogeneous enhancement. **(B)** Portal phase showed persistent enhancement. **(C)** Delayed phase indicated persistent enhancement, and the degree of enhancement was basically the same as that in the arterial phase. Red arrows indicated the mass, yellow arrows showed the gallbladder and intracystic stones, and blue arrows implied the common bile duct. Preoperative abdominal MRI and MRCP imaging. **(D)** Coronal T2WI indicated a mass located at the hepato-duodenal ligament with ovoid in shape and clear borders. The mass was also adjacent to the common bile duct. **(E)** T2WI showed isotropic signals. **(F)** DWI demonstrated isotropic plus slightly hyperintense signals. **(G)** T1WI also showed isotropic signals. **(H)** MRCP showed no dilatation of the bile ducts and no compression. Red arrow indicated the mass and yellow arrow indicated the bile duct.

A 60-year-old woman sought medical attention because of right upper quadrant pain that had persisted for the past three days. This patient reported no nausea, vomiting, fever, jaundice, history of cancer or infectious diseases such as hepatitis or tuberculosis, and no record of prior surgery or trauma. Physical examination did not find jaundice on the skin and sclera. The abdomen was soft but there was tenderness in the right upper abdomen and percussion pain in the right upper abdomen without rebound tenderness and abdominal muscle tension. Laboratory tests showed white blood cell count 9.78 × 10^9^/L, neutrophil percentage 78%, alanine aminotransferase 58 U/L, aspartate aminotransferase 56 U/L and no abnormalities for coagulation tests and digestive tract tumor markers. Enhanced computed tomography (CT) of the abdomen found gallstones and a thickened gallbladder wall with signs of chronic inflammation. The bile duct was not dilated, and an oval mass was detected in the hepatoduodenal ligament, 3.2 × 2.7 × 2.5 cm in size and with clear borders. The arterial phase, portal venous phase and delayed phase all showed uniform enhancement, with no indications of compression or invasion into surrounding organs and tissues (**Figures 1A-C**). Abdominal magnetic resonance imaging (MRI)+magnetic resonance cholangiopancreatography (MRCP) showed that the location of the mass was consistent with the CT findings, with clear boundaries, uneven T1 and T2 signals and slightly high diffusion weighted imaging (DWI) signal. The mass was adjacent to the common bile duct. MRCP showed no signs of compression or dilation of the intrahepatic or extrahepatic bile ducts (**Figures 1D-H**).

**Figure 2:**
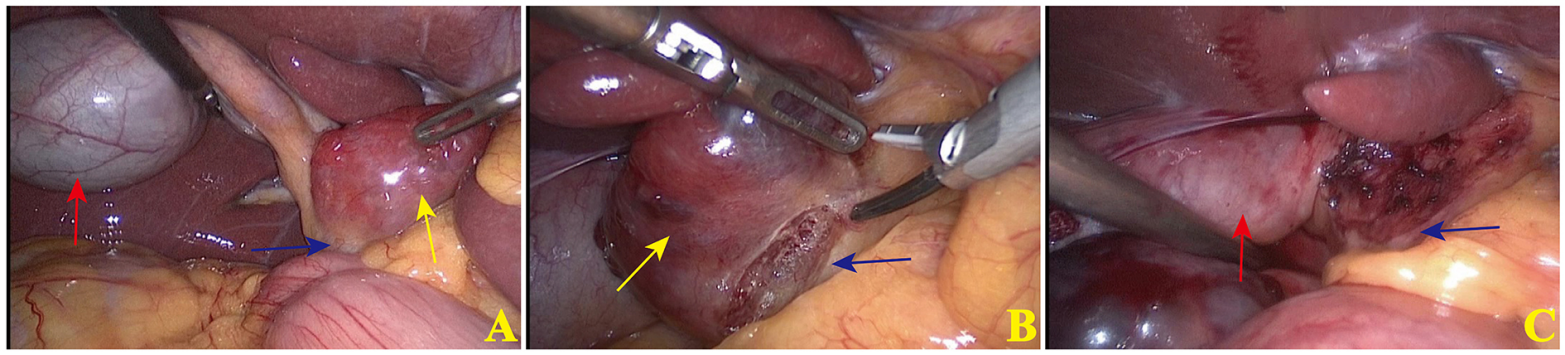
Typical images of surgical procedures. **(A)** Laparotomy was performed, and yellow arrow indicated the mass, red arrow indicated the gallbladder and blue arrow indicated the common bile duct. The mass was located anterior to the hepato-duodenal ligament, and the common bile duct was posterior to the mass. **(B)** Ultrasonic scalpel was applied to resect the mass, which showed the sparse tissues between the wall of the mass and the common bile duct. **(C)** Trauma surface of the mass after complete resection.

**Figure 3:**
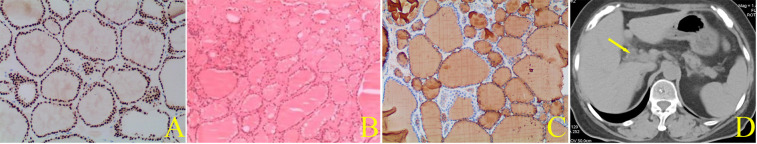
Pathological confirmation of specimen. **(A)** Hematoxylin-eosin staining showed differentiated thyroid tissue (×100). **(B)** Immunohistochemical staining demonstrated that intraoperative specimen was positive for thyroid transcription factor-1 (×200). **(C)** Immunohistochemical staining indicated that intraoperative specimen was positive for thyroglobulin (×200). Postoperative abdominal CT imaging after six-month follow-up. **(D)** Postoperative CT imaging showed alterations after gallbladder and ectopic thyroidectomy. Yellow arrow indicated the common bile duct.

The preoperative laboratory tests revealed abnormal white blood cell, neutrophil counts and abnormal liver enzymes, which were attributed to gallbladder inflammation. Considering the patient’s symptoms and imaging results, the diagnosis of gallbladder stones combined with cholecystitis was confirmed. The patient was advised to undergo fine-needle aspiration to clarify the nature of the mass but this was refused by the patient. Considering the diagnosis of gallstones and cholecystitis, as well as the indication for cholecystectomy, laparoscopic cholecystectomy and exploratory resection of the mass were performed after obtaining the patient’s informed consent. During intraoperative exploration (**Figure 2**), it was observed that the gallbladder displayed signs of chronic inflammation, with palpable stones within. A mass of bright red color and firm texture was located in the hepatoduodenal ligament, in front of the common bile duct. Apart from the posterior attachment of the mass to the hepatoduodenal ligament, the mass was relatively independent and did not exhibit connections with adjacent organs such as the liver, gallbladder and stomach. During the operation, it was discovered that a tissue gap existed at the point where the mass was attached, and there was no invasion of the common bile duct. An ultrasonic knife was used to peel and remove the mass completely along the loose tissue gap between the mass and the duodenal ligament, following which the gallbladder was removed. Postoperative pathology of the gallbladder showed gallstones and chronic cholecystitis. Pathology of the mass showed thyroid tissue, with no obvious epithelial atypia. Immunohistochemical staining showed positivity for thyroid transcription factor-1 and thyroglobulin. Based on the morphological and immunohistochemical results (**Figures 3A-C**), the mass was diagnosed as ectopic thyroid. Subsequent examination of the neck of the patient revealed the presence of a normal thyroid.

The patient was routinely given symptomatic and supportive treatment with anti-inflammatory and hepatoprotective drugs after surgery. Given that there was no abnormality in thyroid function, there was no related treatment. The patient recovered and was discharged seven days after surgery. There were no abnormalities in abdominal CT and thyroid function tests six months after the operation (**Figure 3D**), and there were no abnormalities in the patient’s thyroid ultrasonography and thyroid function during follow-up for the next four years.

## Discussion

It is estimated that the prevalence of ectopic thyroid is about 1:100,000–300,000, and about 1:4000–8000 among patients with thyroid diseases. Given its low incidence and lack of specific clinical manifestations, the condition is often misdiagnosed and inappropriately treated (6). Ectopic thyroid manifests in the following two types. The first type involves the thyroid gland being present in its normal anatomical position in the front of the neck, formed by the normal descent of a portion of the primordial thyroid tissues, in addition to thyroid tissue in other locations, which is formed by the abnormal descent of some primordial thyroid tissue. This is called partial ectopic thyroid, also known as an extra thyroid gland. The second type involves the absence of the thyroid gland in its usual anatomical position. In this scenario, the presence of thyroid tissues in other locations results from the abnormal descent of the entire primordial thyroid, termed complete ectopic thyroid, also known as vagal thyroid (7). There are even instances of double ectopia. In a case, ectopic thyroid glands were identified in both the porta hepatis and the base of the tongue, with no glands detected in the thyroid bed (8). This case involved ectopic thyroid situated within the intra-abdominal hepatoduodenal ligament. The mass showed no apparent association with nearby organs, and the patient had no history of tumor-related diseases. The diagnosis of ectopic thyroid demands careful consideration, and an accurate diagnosis prior to surgery is crucial. This is essential to prevent lifelong complications such as hypothyroidism or the inadvertent loss of thyroid function that may occur if the ectopic thyroid is mistakenly removed.

A comprehensive database search identified a total of 10 reported cases related to ectopic thyroid in the hepatoduodenal ligament and its surrounding areas from 1991 to 2022 (8–17). The case information is summarized in **Table 1**. Importantly, consistent with these above reports, only one case occurred in the female patient. The one male patient was accidentally diagnosed with ectopic thyroid by postoperative pathology after undergoing pancreaticoduodenectomy for pancreatic cancer (16). Moreover, only one patient received endoscopic ultrasonic fine-needle aspiration biopsy, thus avoiding surgical resection (17). All the other patients underwent surgical treatment. Fortunately, these patients all had partial ectopic thyroid, with a normal thyroid and function. Regardless of its size, the ectopic thyroid gland in the hepatoduodenal ligament and surrounding area did not cause symptoms of obstruction of surrounding organs, such as jaundice, pancreatitis, portal hypertension and gastrointestinal obstruction, caused by compression of the bile duct, pancreatic duct, hepatic portal vessels and gastrointestinal tract respectively. Ectopic thyroid gland without the above abnormalities may be considered as normal glandular tissue. The glandular tissue is soft and non-invasive to surrounding tissues or organs. This may explain why ectopic thyroid does not cause symptoms of compression, and it is also a characteristic that distinguishes ectopic thyroid from malignant tumors. Review of these 10 cases suggests that for these masses that are difficult to identify by routine approaches, ectopic thyroid should be considered if characteristics including female sex, clear boundaries, lack of invasiveness and no symptoms of compression and obstruction of surrounding organs are present.

**Table 1:** Summary of ectopic thyroid cases in the hepatoduodenal ligament and its surrounding areas.

Age	Sex	Location	Size (mm)	Surgical resection	Thyroid diseases	Reference	Year
63	Female	First porta hepatis	43×38	Yes	No	(9)	2021
24	Female	First porta hepatis	100×90	Yes	No	(8)	2003
63	Female	First porta hepatis	50	Yes	No	(10)	2019
60	Female	Gallbladder wall	19×17×11	Yes	No	(11)	2010
57	Female	Wall of common bile duct	3×2	Yes	No	(12)	2020
73	Female	Head of the pancreas	60×50	Yes	No	(13)	2017
53	Female	Double gallbladder intra-septal	30×10	Yes	No	(14)	2013
39	Female	Gallbladder wall	Undescribed	Yes	No	(15)	2019
63	Male	Submucosa of descending duodenum	Undescribed	Yes	No	(16)	1991
65	Female	Gallbladder bed	80×55	No	Surgical removal of toxic goitre	(17)	2022

The diagnosis of ectopic thyroid relies not only on the above characteristics but also requires imaging examination, and ultimately is confirmed by pathology. Commonly used imaging examinations include ultrasound, CT and MRI (9). Ectopic thyroid usually appears as a hyperechoic or non-uniform mass under ultrasound. CT and MRI can clearly locate the mass and are helpful for preoperative evaluation. Enhanced CT can show a significantly enhanced mass and plain CT may show a slightly higher signal than the surrounding tissue. The contrast-enhanced CT of this case showed significant enhancement in all three phases, which was consistent with literature reports. However, the characteristics of ectopic thyroid on MRI are diverse, which may be attributed to variation of the colloid material content in ectopic thyroid. Commonly employed examinations lack distinctive features. Thyroid tissue not only takes up the radioisotope, but also this helps in localizing the ectopic thyroid and in determining the presence of a utopic thyroid gland. Radionuclide imaging serves as a valuable diagnostic tool, where thyroid imaging utilizing Tc-99m, I-131 and I-123 can effectively identify ectopic thyroid tissues. Radionuclide imaging can also detect double ectopic thyroid and thyroid in the normal anatomical position, which is important for preoperative evaluation (13). Tc-99m is generally the preferred choice owing to its superior image quality, lower radiation dose, cost-effectiveness compared to I-131, and safety for use in children (18). Needle biopsy is an important diagnostic technique. The pathological diagnosis of ectopic thyroid through needle biopsy offers a significant basis for determination of treatment strategies. However, pathology is the gold standard for the diagnosis of ectopic thyroid (19). Immunohistochemistry can confirm the pathological diagnosis by demonstrating the positivity of thyroid transcription factor-1 and thyroglobulin. Pathological diagnosis by needle biopsy is strongly recommended before treatment, and is especially important in patients with complete ectopic thyroid. In this current case, the patient refused needle biopsy and directly received surgical treatment. Fortunately, subsequent neck CT and thyroid laboratory tests confirmed the presence of normal thyroid and thyroid function.

Ectopic thyroid can be treated on the basis of its type, size, clinical symptoms, severity of the condition, thyroid function, and malignant status. Individualized treatment should be emphasized, and if necessary, a multi-disciplinary treatment group consisting of endocrinologists, radiologists, general surgeons and otolaryngologists should collaboratively make scientifically sound diagnostic and treatment decisions (20). Ectopic thyroid is considered normal tissue, and if clinically asymptomatic and without signs of malignancy, it can be followed up with observation. Surgical options include partial excision, total excision, pedicle transfer and auto-transplantation, primarily addressing cases with severe compressive symptoms. Before proceeding with surgery, it is essential to confirm the presence of normal thyroid. If confirmed, total resection of the ectopic thyroid can be carried out. Otherwise, partial resection can be performed to relieve symptoms of obstruction (21). In cases with signs of malignancy, the scope of resection should be determined based on intraoperative frozen section biopsy. In general, radical surgery is the first choice for ectopic thyroid cancer (22). Regular follow-up is recommended for individuals with ectopic thyroid, regardless of whether they undergo treatment. The follow-up should include imaging and functional tests of both the normal thyroid gland and ectopic thyroid gland. The patient we report underwent postoperative follow up for four years and her thyroid and thyroid function did not show abnormalities.

## Conclusions

In summary, we report the first case of ectopic thyroid situated in the hepatoduodenal ligament and the front of the common bile duct. This case is also the first to showcase intra-abdominal ectopic thyroid through authentic surgical images, offering a more instructive visual representation. Through a review of pertinent literature on ectopic thyroid in the hepatoduodenal ligament and nearby regions, it was aimed to enhance clinicians’ diagnostic and treatment perspectives. It is crucial to emphasize that, in cases of ectopic thyroid, achieving a clear diagnosis before initiating treatment is of great importance.

## Data availability statement

The raw data supporting the conclusions of this article will be made available by the authors, without undue reservation.

## Ethics statement

The studies involving humans were approved by Qingdao University Affiliated Weihai Central Hospital. The studies were conducted in accordance with the local legislation and institutional requirements. Written informed consent for participation in this study was provided by the participants’ legal guardians/next of kin. Written and signed informed consent for anonymous collection and analysis of clinical data as well as publication of relevant data was obtained from the patient. Written informed consent was obtained from the participant/patient(s) for the publication of this case report.

## Author contributions

LZ: Conceptualization, Formal analysis, Writing – original draft, Writing – review & editing. XC: Conceptualization, Writing – review & editing. BW: Writing – review & editing. XD: Writing – review & editing. GH: Writing – review & editing. XY: Writing – review & editing.
